# Human NK Cell Up-regulation of CD69, HLA-DR, Interferon γ Secretion and Cytotoxic Activity by Plasmacytoid Dendritic Cells is Regulated through Overlapping but Different Pathways

**DOI:** 10.3390/s90100386

**Published:** 2009-01-09

**Authors:** Adel Benlahrech, Heather Donaghy, George Rozis, Martin Goodier, Linda Klavinskis, Frances Gotch, Steven Patterson

**Affiliations:** 1 Department of Immunology, Imperial College, Chelsea & Westminster Hospital, London, UK; 2 Department of Immunobiology, King's College London, Guys Hospital, London, UK

**Keywords:** dendritic cells, NK cells, cytokines, cytotoxicity

## Abstract

Human plasmacytoid dendritic cells secrete high levels of IFNα and are thus implicated in the activation of NK cells. Activated NK cells are characterised by the up-regulation of CD69 and MHC class II DR expression, secretion of IFN γ and enhanced cytotoxicity. We show that pDC mediate these processes by different mechanisms, some of which overlap. Human NK cells were analysed after co-culture with immature or CpG-matured blood pDC or with supernatant from these cells. Maximal CD69 expression by NK cells was mediated by supernatant from mature pDC and did not require pDC contact. Up-regulation was due in part to IFNα but also to factors in IFNα negative supernatant from immature DC. HLA-DR expression was independent of secreted molecules but required contact with immature or mature DC. Enhanced NK cytotoxicity, measured by killing of K562 targets and expression of CD107a, was mediated by multiple factors including type I IFN, supernatant from immature pDC cultures and contact with immature or mature pDC. These factors act cumulatively to enhance cytotoxcity. Thus different parameters of pDC mediated NK cell activation are regulated by distinct pathways.

## 1. Introduction

Dendritic cells (DC) and natural killer (NK) cells represent two specialised cell types of the innate immune system [1, 2]. DC are a distinct population of bone marrow derived leukocytes that act as biological sensors able to detect inflammatory cytokines and invading pathogens through a broad range of receptors and then mature and migrate to secondary lymphoid tissue, where they induce antigen-specific naïve T cell activation and proliferation [1, 3]. It is well established that DC *in-vivo* are a heterogeneous population based on phenotype, morphology, and function [4]. In humans, at least two different blood DC populations have been described, based on their phenotype and cytokine secretion profiles [5]. One population, referred to as myeloid DC (mDC), expresses myeloid markers including CD11c, CD13 and CD33 and secretes IL-12 on stimulation by CD40 ligand. The second population lacks myeloid markers, but expresses the receptor for IL-3, CD123, and are potent producers of IFNα on stimulation by viruses [4, 6] or bacterially derived CpG DNA through TLR9 [7]. This latter population differentiate into cells with a plasma cell-like morphology, and hence are termed plasmacytoid DC (pDC) [8]. In addition to their role in innate immune responses pDC can also process and present virus antigen to CD4 and CD8 T cells [9]. The development of the monoclonal antibodies BDCA-1 and BDCA-4, that label mDC and pDC respectively, has greatly facilitated their purification from blood [10].

Natural killer cells were first described as a result of their ability to kill tumour cells without prior sensitisation [2, 11]. Later studies demonstrated that NK cells recognise and kill potentially harmful cells that have lost their MHC class I molecules, including virally-infected and tumour cells, and led to the proposal of the missing-self hypothesis [12-14].

Recent studies have demonstrated interactions between DC and NK cells which result in the maturation of DC and activation of the lytic function of NK cells enabling them to kill immature but not mature DC [15-18]. Due to the low numbers of blood DC (less than 1% of total PBMC) most studies have used *in-vitro* generated monocyte-derived DC (mdDC) [15-17] to study this DC/NK cross-talk. However, care must be taken in extrapolating these findings to the naturally occurring heterogeneous DC populations. This is further emphasised by early studies where Chehimi *et al.* [19] demonstrated that only IFNα producing cells (pDC) provide accessory function required for NK cell mediated lysis of cytomegalovirus-infected target cells, whereas plastic adherent blood DC (myeloid DC) lack this capacity to induce NK lytic activity [19]. Although two independent groups [20, 21] have recently addressed the involvement of pDC in NK cell activation, we have further characterised the extent of pDC-mediated stimulation of several NK cell functions and analysed the mechanisms involved with particular emphasis on the effects of type I IFN and the differential ability of immature versus mature pDC to stimulate NK cells.

## 2. Materials and Methods

### 2.1 Cell isolation (pDC and NK cells)

PBMC from single-donor buffy coat blood packs (National Blood Transfusion Service, UK) were isolated by Histopaque (Sigma-Aldrich, UK) density centrifugation then separated through a 50% percoll (Sigma-Aldrich, UK) gradient (30 min at 300 g). pDC were magnetically isolated from the interface of the percoll gradient using BDCA-4 microbeads (Miltenyi Biotec, Germany) in accordance with the manufacturer's protocol. Autologous NK cells were purified from the lymphocyte fraction at the bottom of the gradient by positive magnetic separation using CD56 microbeads (Miltenyi Biotec, Germany) followed by depletion of NKT cells using anti-CD3 conjugated Dynal beads, and were frozen in FCS containing 10% dimethyl sulfoxide (DMSO) (Sigma-Aldrich, UK) for later use.

### 2.2 Co-cultures

Immature and mature pDC populations were used for co-culture with autologous NK cells. Freshly isolated blood pDC cultured in the presence of IL-3 for 24 hours were used as immature DC. Mature pDC were generated by stimulating freshly isolated blood pDC with 6 μg/mL CpG ODN (G*G*GGGACGATCGTCG*G*G*G*G*G, Oswel, UK) [7] for 24 hours in the presence of IL-3. Previous studies have shown that, compared to pDC maintained in IL-3 alone, pDC stimulated with CpG show a more dendritic morphology and express higher levels of co-stimulatory molecules [22]. pDC were washed before co-culture with autologous NK cells (at a ratio of 1:5) for a further 24 hours. DC-NK cell culture supernatants were removed and stored at -20°C for cytokine assay by ELISA. In some experiments NK cells were incubated with a type I IFN receptor blocking antibody (10 μg/mL, or otherwise indicated) (R&D Systems, UK) for 20 minutes on ice, prior to co-culture with DC. In other experiments, NK cells were incubated for 24 hours with rhIFNα (1 μg/mL, Sigma-Aldrich, UK), or with supernatants from pDC that were cultured in the presence of IL-3, or IL-3 and CpG DNA for 24 hours. As a positive control of activation, NK cells were cultured in the presence of IL-2 (50 units/mL, R&D Systems, Abingdon, UK), influenza virus (H3N2 strain X-31), or with PMA (50 ng/mL) and Ionomycin (1 μM) (both from Sigma-Aldrich, UK).

### 2.3 Phenotypic characterisation

Surface antigen expression was analysed by three or four-colour direct immuno-fluorescence using a fluorescent-activated cell sorter (FACS) (FACS-calibur, BD). The following monoclonal antibodies were used: phycoerythrin (PE) conjugated anti-(CD3-PE); Peridium Chlorophyll-a protein (PerCP) conjugated anti-CD3; anti-CD14-PE; anti-CD19-PE; anti-CD56-PE; fluorescein isothiocyanate (FITC) conjugated anti-CD69; anti-CD11c-FITC; anti-CD69-PerCP; anti-HLA-DR-FITC; anti-HLA-DR-PerCP; Allophyocyanin (APC) conjugated anti-HLA-DR-APC; anti-CD3-APC; and anti-CD56-APC (Becton Dickinson/Pharmingen, Oxford, UK). Cells were pelleted then resuspended in 200 μL of FACS buffer (PBS containing 2% FCS, 2 mM EDTA, and 0.05% NaN_3_), stained on ice with fluorescent antibodies for 30 minutes, washed with FACS buffer and then fixed with 4% parafomaldehyde in PBS. Appropriate isotype control antibodies were used to assess the level of specific labelling.

### 2.4 Intracellular detection of IFNγ

NK cells alone or with DC were incubated for 24 hours at 37°C. Brefeldin-A (10 μg/mL, Sigma-Aldrich, Poole, UK) was added for the last 5 hours of culture. Cells were then fixed with 2% paraformaldehyde in PBS for 15 minutes at room temperature, washed and stained for 20 minutes at room temperature with anti-CD56-PE, anti-CD69-PerCP, anti-HLA-DR-PerCP or anti-CD3-PerCP, and anti-hIFNγ-FITC (BD Pharmingen, Oxford, UK) antibodies in the presence of 0.5% saponin. The cells were washed and fixed in 4% paraformaldehyde before being analysed by FACS.

### 2.5 Detection of CD107a on NK cells

The percentage of degranulating NK cells was measured as previously described by Alter *et al.* [23]. Briefly, NK cells were incubated alone, or with immature or mature DC either cultured together or separated by 4 μm transwells (R&D Systems), or treated with immature or mature pDC supernatants for 24 hours. Cells were then harvested and incubated with K562 cells for 4 hours at an E:T ratio of 5:1 in the presence of monensin (6 μg/mL, Sigma-Aldrich, UK) and anti-human CD107a-FITC antibody (BD Pharmingen, Oxford, UK). Cells were then surface labelled with anti-CD3-PerCP and anti-CD56-APC antibodies (BD Pharmingen). The cells were washed and fixed in 4% paraformaldehyde before being analysed by FACS.

### 2.6 ELISA assays

The levels of IFNα, IFNγ, and IL12_p70_ in DC/NK cell co-culture supernatants were quantified by sandwich ELISA. IFNα was detected using monoclonal mouse-anti-human IFNα antibody (R&D Systems, UK), polyclonal sheep anti human IFNα antibody (R&D systems, UK), and rabbit-anti-sheep antibody-HRP (Dako, Ely, UK). IFNγ was measured using IFNγ matched antibodies (R&D systems, UK) in accordance with the manufacturer's protocol. IL-12_p70_ was detected using rat anti-human IL-12_p70_ antibody (BD Pharmingen), biotinylated-mouse-anti-human IL-12_p40/70_ antibody (BD Pharmingen), and horseradish peroxidase avidin D (Vector Laboratories, Peterborough, UK). The optical absorbance of the ELISA plates was read at 405nm and cytokine levels were calculated from values obtained using standard curves determined from recombinant cytokines (rhIFNα, Sigma-Aldrich, UK; rhIFNγ, R&D systems, UK; IL-12_p70_, Beckton Dickinson, UK).

### 2.7 Cell mediated cytotoxicity assay

NK specific lysis of K562 cells was measured by non-radioactive cytotox96 assay (Promega, Southampton, UK) according to the manufacturer's protocol. In brief, NK cells were incubated alone, or with immature or mature DC either cultured together or separated by 4μm cell culture inserts (R&D Systems), or treated with immature or mature pDC supernatants for 24 hours. Cells were then harvested and incubated with K562 cells at an E:T ratio of 5:1 for 4 hours. Lactate dehydrogenase (LDH) levels were then quantified. The percentage of specific cytotoxicity was then calculated according to the manufacturer's protocol.

## 3. Results

### 3.1 Purity of the pDC and NK populations

**Figure 1.**
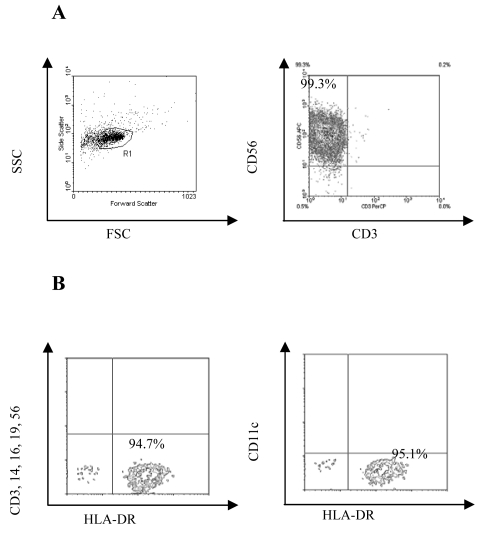
Purity of the different isolated cell populations. A) NK cells were isolated as described in materials and methods then stained for CD56 and CD3. B) autologous pDC were magnetically isolated then stained with anti-human CD11c, a cocktail of anti-human CD3, CD14, CD16, CD19, and CD56 and HLA-DR antibodies. Contour plots are expressed with a threshold value of 0.2; percentages of purity were obtained using CellQuest Pro^®^.

**Figure 2.**
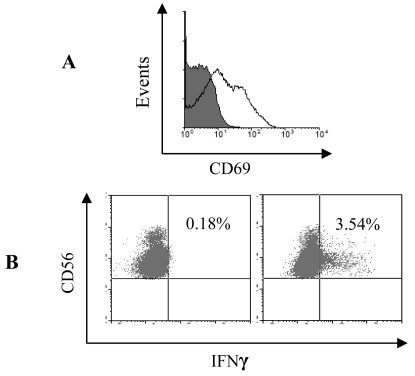
NK activation after a single freeze/thaw cycle. Isolated NK cells were cryopreserved and re-thawed for use in later experiments. A) NK cells cultured for 24 hours in the absence (filled histogram) or presence of IL-2 (clear histogram) were labelled with anti-CD56, CD3 and CD69 antibodies. Histograms show CD69 expression on CD56^+^ CD3^-^ cells. B) NK cultured alone for 24 hours (left dot-plot) or with IL-2, 50 units/mL, (Right dot-plot) and stained for intracellular IFNγ. Dotplots show 50,000 acquired events gated on live CD56^+^ CD3^-^ cells.

NK cells were magnetically isolated based on CD56 expression followed by magnetic depletion of NKT cells using anti-CD3 conjugated Dynal beads (Figure 1A). The isolation process did not induce NK activation as determined by the low expression of the early activation marker CD69 and flow cytometric detection of intracellular IFNγ (Figure 2) after 24 hours of culture. The isolated NK cells were frozen for later use in co-culture experiments with pDC. The functional viability of stored NK cells was confirmed by stimulating with IL-2 [24, 25] (Figure 2) and influenza virus [26] (data not shown) and demonstrating upregulation of CD69 expression and production of IFNγ. pDC were isolated using BDCA-4 magnetic beads. Their purity was evaluated by fluorescent-activated cell sorter (FACS) based on their lack expression of lineage-specific markers (CD3, CD14, CD16, CD19, CD56), high expression of HLA-DR molecules, and absence of CD11c expression. The purity of the preparations varied from 87-99%. A representative phenotypic profile of pDC is depicted in Figure 1B.

### 3.2 pDC induce CD69 up-regulation on NK cells in a cell-contact independent, type I interferon dependent mechanism

**Figure 3.**
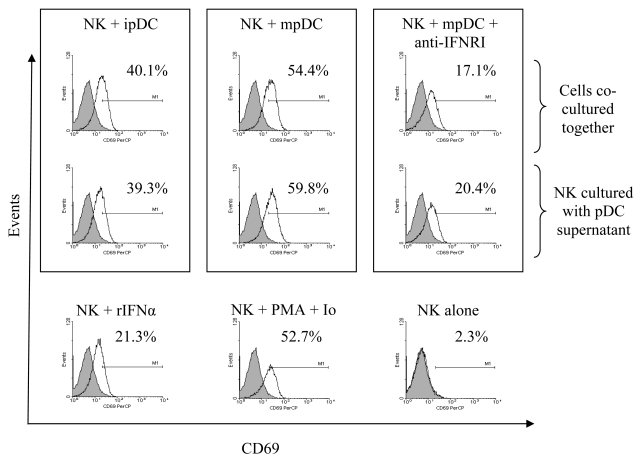
NK cells up-regulate CD69 in response to pDC sensitisation through secreted type I interferons. NK cells were cultured for 24 hours in the presence or absence of anti-IFNR1 antibody. Cells were cultured alone or treated with PMA/Ionomycin, rIFNα, untreated (ipDC) or CPG-treated pDC (mpDC), or their supernatants. Cells were then stained with anti-CD3, CD56, CD69 and HLA-DR antibodies and CD69 expression shown on the CD3^-^ CD56^+^ cells (clear histograms). Results from a typical experiment (total of 3) are presented with M1 region based on the isotype control (filled histograms).

We first assessed the capacity of pDC to activate autologous NK cells by flow cytometry. Prior to co-culture with NK cells pDC were cultured for 24 hours with IL-3 or with IL-3 and CpG DNA to induce maturation [22]. Co-culture of NK cells with pDC at a ratio of 5:1 resulted in up-regulation of CD69 on NK cells. Activation of NK cells was mediated by both immature and mature pDC with a more pronounced induction by mature DC (Figure 3).

We next investigated whether pDC-mediated up-regulation of CD69 on NK cells was mediated by secreted factors, such as type I interferons, or whether direct cell-cell contact was essential for such effect. NK cells were either directly co-cultured with immature or mature pDC for 24 hours, or treated with supernatants from immature or mature pDC for the same duration of time. (Note that CpG stimulated pDC continue to secrete IFNα after 24h as shown in Figure 5). Cultures were performed in the presence or absence of anti-human IFN type 1 receptor (IFNAR1) antibody. No significant differences were found in the levels of CD69 up-regulation by NK cells in response to either pDC or their supernatants, indicating that this effect is solely mediated by secreted factors. Pre-treatment of NK cells with anti-IFNR1 antibody significantly reduced but did not ablate CD69 expression on NK cells (Figure 3). Culture of NK cells with recombinant IFNα induced CD69 up-regulation but the level of expression was less than that observed with supernatant from immature or mature pDC. Taken together, these results indicate that pDC mediate CD69 up-regulation on NK cells via signalling through the type I interferon receptor, by a non type I IFN component released by immature and probably by mature pDC, and is cell contact independent.

### 3.3 pDC induce upregulation of HLA-DR on NK cells through cellular contact

**Figure 4.**
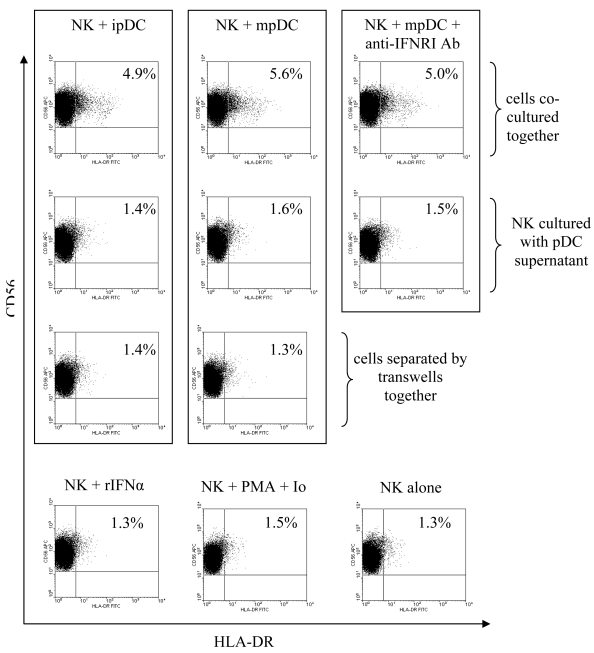
pDC induce HLA-DR up-regulation on NK cells through cellular contact. NK cells up-regulate HLA-DR in response to pDC sensitisation in a cell–contact dependent, type I interferon independent manner. NK cells were cultured for 24 hours in the presence or absence of anti-IFNR1 antibody. Cells were cultured alone or treated with PMA/Ionomycin, rIFNα, untreated (ipDC) or CPG-treated pDC (mpDC), or their supernatants. NK cells were cultured with pDC either together, or separated by transwells. Cells were then stained with anti-CD3, CD56, CD69 and HLA-DR antibodies. HLA-DR expression on the CD3^-^ CD56^+^ cells is shown. Results from a typical experiment (total of 3) are presented with quadrants based on the isotype control.

We further investigated the extent of NK cell activation by measuring the levels of CD18, a β_2_-integrin [27, 28], and HLA-DR [29, 30] up-regulation upon pDC sensitisation. Again, pDC were incubated with IL-3, or IL-3 and CPG for 24 hours prior to being co-cultured with autologous NK cells at a ratio of 1:5 respectively. Co-culture of NK cells with pDC resulted in no changes in the levels of CD18 expression on NK cells (data not shown). Interestingly, we observed up-regulation of HLA-DR on NK cells in response to pDC stimulation (Figure 4). The levels of HLA-DR upregulation on NK cells were similar in response to either immature or mature pDC. We next examined whether this activation is driven by pDC-secreted factors or by direct cell-cell contact. Mature or immature pDC were cultured with NK cells or separated by transwells and anti-IFNRI antibody was added to some cultures. NK cells were also treated for 24 hours with supernatant from immature or mature pDC in the presence or absence of anti-IFNRI antibody. Unlike CD69 expression, HLA-DR up-regulation was restricted to NK cells that were allowed cell contact with pDC (Figure 4) and was not inhibited by anti-IFNRI antibody. Furthermore, NK cells treated with rIFNα for 24 hours failed to up-regulate HLA-DR. Thus pDC-mediated HLA-DR up-regulation on NK cells is type I interferon independent, cell contact dependent.

### 3.4 Mature pDC induce IFNγ secretion by NK cells via signalling through the type I interferon receptor

**Figure 5.**
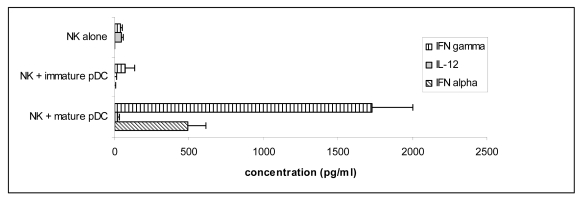
Mature pDC induce IFNγ secretion by NK cells.

NK activation was further analysed by measuring IFNγ release in co-culture supernatants. Unlike CD69 or HLA-DR induction, only mature pDC were found to induce high levels of IFNγ secretion by NK cells (Figure 5). As expected in cultures of pDC induced to mature with CpG DNA, we also observed secretion of high levels of IFNα [6, 31].

**Figure 6.**
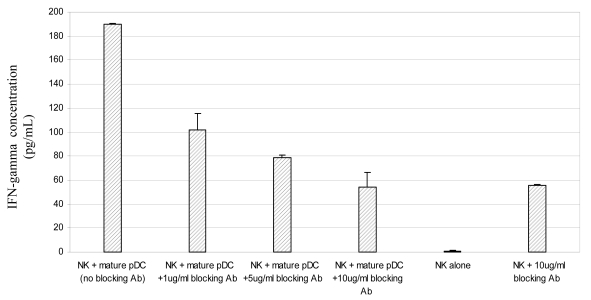
pDC stimulate NK secretion of IFNγ and is dependent on signalling through the type 1 interferon receptor.

To determine whether the activation of NK cells by pDC is mediated by type 1 interferon the co-culture experiments were repeated in the presence of increasing amounts of anti- IFNAR1 antibody. Although both the antibody (Figure 6) and its isotype control (data not shown) induced IFNγ secretion by NK cells cultured alone, probably by sensitisation through CD16 Fc receptor [32], the levels of IFNγ released by NK cells cultured with CpG-matured pDC were three-fold higher. Addition of the anti-IFNR1 antibody to co-cultures of mature pDC and NK cells inhibited production of IFNγ by NK cells in a dose-dependent manner (Figure 6).

### 3.5 NK cell degranulation is induced by type I interferons and enhanced by cellular contact with pDC

**Figure 7.**
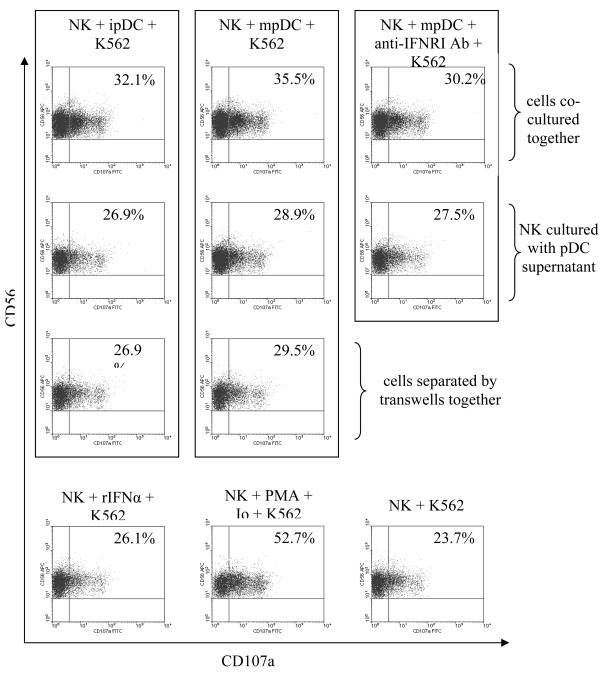
NK degranulation is slightly induced by type I interferons but augmented through cellular contact with pDC.

Recently, lysosomal-associated membrane protein-1 (LAMP-1 or CD107a) was shown to be a marker for NK cytolytic activity [23] Therefore, we assessed whether CD107a expression was up-regulated on NK cells following stimulation with pDC. NK cells were co-cultured with immature or mature pDC for 24 hours then incubated with K562 tumour cells for 4 hours in the presence of monensin and anti-human CD107a antibody. Stimulation of NK cells with either immature or mature pDC increased the percentage of CD107a^+^ NK cells compared to basal CD107a expression on NK cells cultured with K562 cells (Figure 7). The induction of CD107a expression was more pronounced when NK cells were stimulated by mature pDC.

To determine whether the increased degranulation by NK cells is mediated through cellular contact or through soluble factors, NK cells were treated with supernatants from immature or mature pDC in the presence or absence of anti-IFNR1 antibody, or separated from pDC by transwells. NK cells that were separated from pDC or treated with pDC supernatants showed a slight increase in the levels of CD107a expression (Figure 7). This increase, however, was less than what was observed in NK cells that were allowed direct contact with pDC. Furthermore, blocking the IFNR1 receptor on NK cells only resulted in a small decrease in the percentage of CD56^+^ CD107a^+^ cells. This reduction was consistent with the difference between the effect of immature and mature pDC. Moreover, treatment of NK cells with rIFNα resulted in a small increase in the percentage of CD107a expressing NK cells that was also consistent with the difference between the effect of immature and mature pDC on NK cell degranulation. Thus pDC mediate degranulation of NK cells in response to K562 cells by two mechanisms. First by secreting type I interferons and second, and more predominantly, through a cell-contact dependent mechanism.

### 3.6 Activation of lytic function by NK cells is enhanced by IFNα, but mainly driven by direct contact with pDC

**Figure 8.**
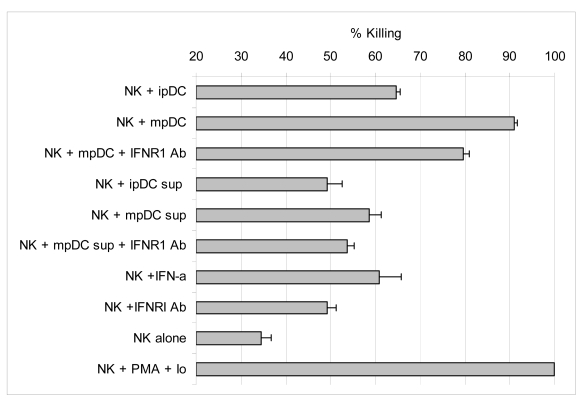
NK specific cytotoxicity is induced by pDC in a contact dependent manner.

We next determined whether NK cells directly co-cultured with pDC could mediate lysis of the tumour cell line K562. The cytotoxicity of NK cells was measured by LDH release from K562 target cells over a 4h period. Both immature and mature pDC were able to prime NK cells to kill K562 tumour cells with high efficiency (Figure 8).

We then asked whether priming for killing required direct cell-cell contact between DC and NK cells. To investigate the requirement for contact and the role of type I interferons, NK cells were treated with immature or mature pDC supernatants in the presence or absence of anti-IFNR1 antibody. Lysis of target cells was enhanced by NK cells treated with pDC supernatants but was substantially less that observed by co-culture with pDC. Furthermore, treatment of NK cells with rIFNα augments NK specific lysis of K562 to a similar extent as that observed in NK cells that were treated with mature pDC supernatant. Treatment of NK cells with anti-IFNR1 antibody slightly activated their lytic activity, probably by non-specific sensitisation through CD16 Fc receptor. Nevertheless, blocking the type I interferon receptor on NK cells causes some reduction in killing. Although we and others [33-35] have established the effect of type I interferon on augmenting NK cytotoxicity, a much greater effect was observed by co-culture of NK cells with pDC. Indeed, contact with either immature or mature pDC led to between 2-3-fold increase in NK-mediated killing whilst blocking the IFNR1 receptor reduced killing to a much lesser extent. These results indicate that pDC enhance the cytotoxic activity of NK cells partly through secretion of type I interferons, but is mainly driven by direct NK-pDC contact.

## 4. Discussion

Multiple changes are seen in NK cells as they are activated to mediate innate immune responses and include the up-regulation of CD69, HLA-DR, secretion of IFNγ and enhanced cytotoxicity. Here we confirm recent findings that human pDC induce NK cell activation [20, 21] but show that the different parameters by which activation is assessed are mediated by different pathways. Type I interferons have long been known to play a role in NK cell activation, but their contribution to all the changes seen on activated NK is unclear. Although pDC produce large amounts of IFNα, this only accounts for some of the changes seen during NK cell activation. Up-regulation of CD69 is an early marker of NK cell activation and does not require contact with the pDC but is due to secreted products although not only IFNα. Three lines of evidence implicate type I interferon. First IFN α secretion was detected in supernatant from mature but not immature pDC correlating with higher CD69 expression induced by mature pDC. Second antibody against the type I interferon receptor reduced CD69 expression induced by mature pDC. Third recombinant IFNα induced expression of CD69 on NK cells but the levels were not as high as that observed with supernatants from immature or mature pDC. The observation that supernatant from immature pDC up-regulates CD69 and the fact that substantial expression remains after blocking the IFN type I receptor suggests that other secreted factors contribute to the up-regulation of CD69. TNFα has been implicated in the upregulation of CD69 by mature pDC [20;21]. Although supernatants from immature pDC were not analysed for this cytokine, co-culture of pDC with NK cells in the presence of a neutralising antibody against TNFα had no effect on the ability of immature pDC to induce CD69 upregulation by NK cells (data not shown).

The ability of pDC to sense NK cells and induce up-regulation of HLA-DR was solely mediated by a cell contact dependent mechanism. Up-regulation was not observed when NK cells were separated from pDC by transwells or when treated with supernatant from mature or immature pDC and expression was not reduced by IFN type I receptor blocking antibody. The observation that both immature and mature pDC stimulated HLA-DR expression suggests the mechanism does not involve interactions between NK cells and co-stimulatory molecules. The possibility that the signal for HLA-DR may have come from pDC/NK clusters was ruled out by lymphocyte gating and exclusion of the pDC population based on expression of high levels of HLA-DR. However, it is plausible that up-regulation of HLA-DR by NK cells in response to direct co-culture with pDC may have been due to a cell-contact dependent transfer of HLA-DR molecules from DC to NK cells. This phenomenon has been previously described in DC-T cell cultures in mice [36, 37]. Nonetheless, it was previously shown that a sub-population of NK cells can function as professional APC presenting conventional antigen [30] and superantigen [29] to T lymphocytes. We propose a model by which pDC stimulate T cell responses, first by direct presentation of antigen to T cells [9], second by augmenting IFNγ secretion by NK cells, which will feed back on T cell responses, and third by increasing the proportion of HLA-DR bearing NK cells which will in-turn stimulate antigen specific T cells.

The capacity of NK cells to secrete IFNγ, an antiviral cytokine [38, 39], in response to pDC sensitisation was found to be dependent on type I interferon. The evidence for this is that only CpG matured but not immature pDC were able to induce NK cell production of IFNγ and was inhibited by antibody to the type I interferon receptor. It has been previously suggested that IFNα enhances NK cytotoxicity but inhibits early NK cell IFNγ production in the presence of functional signal transducer and activators of transcription (STAT) 1 [40]. Here we observe activation of NK cell lytic and IFNγ secreting pathways by CpG-treated pDC that produce IFNα. A possible reason for such discrepancy may be the involvement of other factors, soluble or cell bound, that over-ride the inhibiting capacity of IFNα, or promote IFNα-dependent IFNγ production. In this system we were able to rule out the involvement of IL-12 in mediating NK secretion of IFNγ, in contrast to previous data [41].

To evaluate induction of NK cytotoxic capacity by pDC two different methods were employed, degranulation and killing of the K562 the tumour cell line. In agreement with other investigations, we have found that IFNα can enhance both NK up-regulation of CD107a, a degranulation marker, and NK-specific lysis of K562 cells [7, 42, 43]. Surprisingly, unstimulated immature pDC were found to significantly enhance both degranulation and the lytic function of NK cells against K562 cells. Thus there is a dichotomy between activation of NK cells for lytic activity and secretion of IFNγ. We found that multiple factors act additively to enhance killing. Recombinant IFNα and supernatant from mature pDC enhanced killing but this was less than that observed by co-culture with mature pDC. Killing was reduced by type I IFN receptor blocking antibody but was still substantial. Supernatant from immature pDC also enhanced lytic activity but again this was less than by co-culture with immature pDC. As with the up-regulation of CD69 these findings implicate a secreted product that is not IFNα. Thus pDC enhancement of NK killing is mediated by type I interferon, contact with either immature or mature pDC and a secreted factor that is not IFNα. Preliminary data suggest that this secreted factor is not TNFα, as NK cells co-cultured with immature pDC in the presence of a neutralising antibody against TNFα did not abolish the ability of immature pDC to induce NK cell degranulation (data not shown). In a different system CpG-treated PBMC but not untreated cells mediated lytic activity against K526 targets [7], findings that appear to contradict the activation of NK cell lytic activity that we observed on co-culture with immature pDC. CpG has been shown to enhance the dendritic morphology and co-stimulatory molecule expression by pDC [22] compared with cells cultured with IL-3 alone. However, there is nevertheless some increase in these maturation markers during culture with IL-3 in the absence of CpG [44] and this may explain the differences between our findings and those of Krug *et al.* [7]. IFNα treatment of mdDC has been shown to up-regulate expression of MICA/B, the ligand for NKG2D, the NK cell activating receptor [45]. Since we observed activation of NK cell lytic activity in the absence of IFNα it is not clear whether signalling through these molecules plays a role in stimulation of NK cells by pDC.

A study similar to ours was reported by Gerosa *et al.* [20], but in contrast to our findings, they found that activation of NK cytotoxic activity was contact independent and type I interferon dependent. Several possible factors may explain this discrepancy. First, the type of target cell line used to assess NK cytolytic activity. We used K562 as target cells which are highly sensitive to NK cell killing whereas Gerosa *et al.* utilised the more NK-insensitive Daudi cell line [46]. Furthermore, several reports [47, 48] have indicated that Daudi cells are highly susceptible to IFNα-induced apoptosis, which makes them a less favourable target to use when studying the effect of pDC on NK cells. K562 cells, on the other hand, are more resistant to IFN-α induced apoptosis [49]. The studies also differ in the type of stimulus used to mature the pDC. In the present study, we have avoided the use of influenza virus to induce pDC maturation as we and others [26] have found that the virus directly activates NK functions. Moreover, we have found that stimulation of pDC with influenza virus results in much higher IFNα secretion as compared with CpG DNA (data not shown). Gerosa *et al.* [20], demonstrated that neutralizing IFNα/β in NK/ FLU-virus-activated pDC co-cultures inhibited the pDC mediated NK lysis of Daudi cells by almost half, whereas NK cells that were treated with virus-activated pDC supernatant completely lost their ability to kill Daudi cells upon neutralisation of type I interferons. This indicates that although pDC may activate the lytic function of NK cells through type I interferons, they can also induce NK cytotoxicity through an IFNα independent mechanism as demonstrated by our work. This is in agreement with earlier work conducted by Feldman *et al.* [50] where accessory cells (blood DC) were found to mediate NK lysis of herpes simplex virus-type 1 (HSV-1)-infected cells in both an IFNα-dependent and IFNα-independent mechanisms.

Although there is still controversy regarding the requirement for maturation of mdDC in the activation of NK cells, previous studies [15, 17] have shown that NK cells are able to kill immature but not mature mdDC. This has been proposed as a mechanism to regulate an ongoing immune response. As yet there is no data available regarding the sensitivity of pDC to lysis by NK cells.

In conclusion, we show that NK cell upregulation of CD69, HLA-DR, IFNγ secretion and lytic activity stimulated by pDC is mediated by different pathways some of which are overlapping. Of note, we show, for the first time, that immature pDC are capable of regulating several NK functions including expression of CD69 and HLA-DR and mediating NK cytolytic machinery. However, future challenges include identification of the pDC membrane molecules involved in NK cell activation and characterisation of the activating products secreted by immature pDC. In line with the identification of pDC membrane receptors involved in NK cell activation, it was found in a recent study [51] that culture of immature pDC in the presence of IL-3 resulted in up-regulation of surface expression of glucocorticoid-induced tumour necrosis factor receptor-ligand (GITRL), which in turn resulted in stimulation of NK cell lytic function in a GITRL/GITR dependent fashion. As for the secreted factors by immature pDC that were responsible for stimulating NK cell functions, we were able to rule out in the current investigation the involvement of IL-12_p70_, IFNα, and TNFα in immature pDC-mediated activation of NK cells. However, future studies should perform detailed analysis of the cytokine secretion profile of immature pDC cultured in IL-3, possibly by the means of microarrays, in order to shed light on any potential factors by which these cells can stimulate NK cell functions.
